# Psychosomatic therapy for patients frequently attending primary care with medically unexplained symptoms, the CORPUS trial: study protocol for a randomised controlled trial

**DOI:** 10.1186/s13063-019-3913-3

**Published:** 2019-12-09

**Authors:** Margreet S. H. Wortman, Johannes C. van der Wouden, Janneke P. C. Grutters, Bart Visser, Willem J. J. Assendelft, Henriëtte E. van der Horst, Tim C. olde Hartman

**Affiliations:** 1 ACHIEVE – Centre of Applied Research, Faculty of Health, Amsterdam University of Applied Sciences, Amsterdam, The Netherlands; 2 Department of General Practice and Elderly Care Medicine, Amsterdam UMC, Vrije Universiteit, Amsterdam, The Netherlands; 3 Department for Health Evidence, Radboud University Medical Center, Nijmegen, The Netherlands; 4 Department of Primary and Community Care, Radboud University Medical Center, Nijmegen, The Netherlands

**Keywords:** Psychosomatic therapy, Study protocol, Primary care, Randomised controlled trial, Medically unexplained symptoms, Cost-effectiveness

## Abstract

**Background:**

Medically unexplained symptoms (MUS) are highly prevalent and pose a burden both on patients and on health care. In a pilot study psychosomatic therapy delivered by specialised therapists for patients with MUS showed promising results with regard to patient’s acceptability, feasibility and effects on symptoms. The aim of this study is to establish whether psychosomatic therapy by specialised psychosomatic exercise therapists is cost- effective in decreasing symptoms and improving functioning in patients who frequently consult their general practitioner (GP) with MUS.

**Methods:**

A randomised effectiveness trial with an economic evaluation in primary care with 158 patients aged 18 years and older who are frequently consulting their GP with MUS. Patients will be assigned to psychosomatic therapy in addition to usual care or usual care only. Psychosomatic therapy is a multi-component and tailored intervention, aiming to empower patients by applying psycho-education, relaxation techniques, mindfulness, cognitive approaches and/or graded activity. Patients assigned to the psychosomatic therapy receive 6 to 12 sessions of psychosomatic therapy, of 30–45 min each, delivered by a specialised exercise or physical therapist.

Primary outcome measure is patient-specific functioning and disability, measured with the Patient-Specific Functional Scale (PSFS). Secondary outcome measures are symptom severity, consultation frequency and referrals to secondary care, patient satisfaction, quality of life and costs. Assessments will be carried out at baseline, and after 4 and 12 months.

An economic evaluation alongside the trial will be conducted from a societal perspective, with quality-adjusted life years (QALYs) as outcome measure. Furthermore, a mixed-methods process evaluation will be conducted.

**Discussion:**

We expect that psychosomatic therapy in primary care for patients who frequently attend the GP for MUS will improve symptoms and daily functioning and disability, while reducing consultation frequency and referrals to secondary care. We expect that the psychosomatic therapy provides value for money for patients with MUS.

**Trial registration:**

Netherlands Trial Register, ID: NL7157 (NTR7356). Registered 13 July 2018.

## Introduction

Medically unexplained symptoms (MUS) have been defined as symptoms of which the origins remain unclear after adequate history taking, physical examination and careful consideration of the psychosocial context by a physician [1]. These symptoms are common in primary and secondary care and are often accompanied by psychological, psychiatric and social(−economic) problems. Verhaak et al. estimated that 2.5% of the patients attending primary care can be classified as having persisting MUS [2]. Patients with persistent MUS suffer from their symptoms, are functionally impaired, have a lower quality of life and are at risk of undergoing unnecessary and possibly harmful tests, referrals and treatments [3, 4]. Therefore, these symptoms pose a major burden on patients and society with large societal costs, health care costs and costs of lost productivity [5]. Patients are often dissatisfied with the medical care they receive. They report that they experience a lack of empathy and support, feel stigmatised and not taken seriously, and are worried because neither they nor their physicians understand where the symptoms come from [6, 7].

General practitioners (GPs) often experience patients with MUS as difficult to manage. While many GPs consider MUS to be an expression of psychological distress, patients do not always see the connection between their symptoms and distress. GPs also experience problems in providing plausible explanations for the origin of the symptoms to their patients [8].

The mismatch between perceptions of GPs and patients as described above explains why patients with MUS are often dissatisfied with the medical care that they receive. Limited consultation time, lack of skills of the GP and patients’ resistance towards psychosocial attributions contribute to these difficulties [9].

In primary care, 10% of the patients consulting their GP account for between 30 and 50% of the consultations [10]. Compared to ‘normal’ attenders, these frequent attenders (FAs) generate five times as many prescriptions and hospital contacts and the mean number of GP consultations of persistent FAs compared to non-frequent attenders are seven-fold higher (10.2 and 1.4 visits per year, respectively) [11]. While a proportion of the FAs has one or more diseases that require frequent monitoring, a substantial number of FAs seek medical care for somatic symptoms not explained by physical disease (i.e. MUS) [12].

In the light of the high functional impairment that many of these patients experience, the risks of unnecessary diagnostic procedures and treatments, the problems that GPs face in the management of these patients and the high societal costs one might expect that evidence-based treatment strategies in primary care already exist. However, evidence on effectiveness of various pharmacological and psychological interventions in reducing symptoms and limitations is limited [13]. Several treatments for patients with MUS have been described. Specific interventions for patients with MUS applied in various settings are of limited value for primary care. A Cochrane review assessing the effects of non-pharmacological interventions for MUS concluded that when all psychological therapies included in the review are combined they seem to be superior to usual care or waiting list controls in terms of reduction of symptom severity [14]. However, effect sizes were small and only cognitive behavioural treatment (CBT) had been adequately studied as single treatment. Therefore, the review only allows tentative conclusions for daily practice. Moreover, most patients do not accept CBT as treatment for their MUS. The authors of the review state that the number of studies investigating treatment modalities other than CBT needs to be increased and that this is especially relevant for physical therapies [14].

Psychosomatic therapy is such a physical (multi-component) treatment, administered by physical and exercise therapists with special interest in MUS. It is a stepped-care and tailored approach in which (psycho)-education, relaxation therapy, mindfulness, cognitive behavioural therapeutic interventions and activating exercise therapy are key elements [15, 16].

The therapist explores and treats somatic symptoms by integrating the physical, cognitive, emotional, behavioural and social dimensions of health problems. The therapist explores underlying beliefs and psychosocial factors, which may influence the perceived somatic complaints, with the aim to give patients insight into the interaction of these factors with the somatic complaints and thus to empower patients to regain control over their own health.

Aspects of approaches incorporated in psychosomatic therapy have been shown to be effective in several studies [17–19]. A combination of cognitive behavioural intervention or psycho-education on one hand and exercise therapy on the other was found to reduce pain and fatigue and to increase physical functioning and quality of life in patients with MUS [20]. Our recent pilot randomised trial on the feasibility and treatment effects of psychosomatic therapy showed promising results with regard to patients’ acceptability, feasibility in daily GP practice and effects on symptoms [21]. A Dutch observational before-and-after cohort study by van Ravensberg and van Berkel in 119 patients with MUS demonstrated that patients improve significantly after psychosomatic therapy on self-rated symptom severity, symptoms of distress, quality of life, level of functioning, sick leave and use of medication [22].

Given the abovementioned challenges and lack of evidence, expanding the evidence base for cost-effective approaches in primary care is urgently needed. In order to guarantee optimal health care for patients who frequently attend with MUS in primary care, we aim to study the cost-effectiveness of psychosomatic therapy.

We will evaluate the effects and costs of psychosomatic therapy in primary care for patients who frequently attend the GP for MUS in improving symptoms and daily functioning and disability, while reducing consultation frequency and referrals to secondary care.

In addition to the quantitative (cost-)effectiveness study, we aim to perform a mixed-methods process evaluation to: (1) identify which treatment elements are actually applied; (2) identify the most effective elements of psychosomatic therapy; (3) understand which specific patients can benefit from this approach and (4) explore patients’ experiences with psychosomatic therapy.

## Methods: design, participants, intervention and outcomes

### Study design

**Fig. 1:**
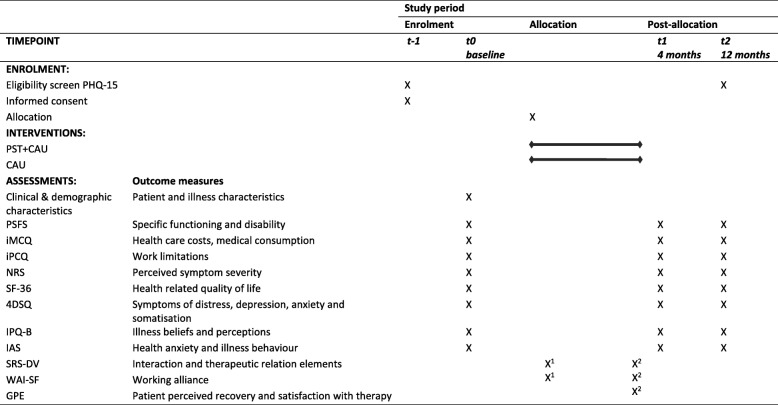
Schedule of enrolment, interventions and assessments. *PST* psychosomatic therapy; *CAU* care as usual; *PHQ-15* Patient Health Questionnaire 15-item somatic symptom scale; *PSFS* Patient Specific Functioning Scale; *iMCQ* iMTA Medical Consumption Questionnaire; *iPCQ* iMTA Productivity Cost Questionnaire; *NRS* numeric rating scale; *SF-36* Short Form Health Survey-36 items; *4DSQ* Four Dimensional Symptom Questionnaire; *GPE* Global Perceived Effect; *IAS* Illness Attitude Scale; *IPQ-B* Brief Illness Perception Questionnaire; *SRS-DV* Session Rating Scale Dutch version; *WAI-SF* Working Alliance Inventory short form; *1* only intervention group, directly after first session; *2* only intervention group directly after last session

The development of this protocol is in accordance with the Standard Protocol Items, Recommendations for Interventional Trials guideline (SPIRIT 2013) [23]. For the SPIRIT Figure see Fig. 1 and for the SPIRIT Checklist see Additional file 1.

We will perform a pragmatic randomised effectiveness trial with economic evaluation in primary care with patients aged 18–80 years who frequently, i.e. twice or more in the past months, consult their GP for MUS. Details regarding recruitment are presented below (see ‘Recruitment of study participants’ section). Patients will be individually randomised into intervention (psychosomatic therapy in addition to usual care) or control condition (usual care alone). The intervention (psychosomatic therapy) consists of 6 to 12 sessions, depending on number and severity of the patient’s symptoms, each of 30–45 min. Therapy will be delivered by a qualified psychosomatic therapist. The primary outcome measure will be functioning and disability measured with the Patient-Specific Functional Scale (PSFS) [24]. All patients will be followed for 1 year and will be asked to complete questionnaires at baseline and at 4 and 12 months’ follow-up. Participants in the intervention group will be referred to one of the 26 participating qualified psychosomatic therapists, physiotherapists or exercise therapists with a special interest in MUS, who receive a training for this study.

**Fig. 2:**
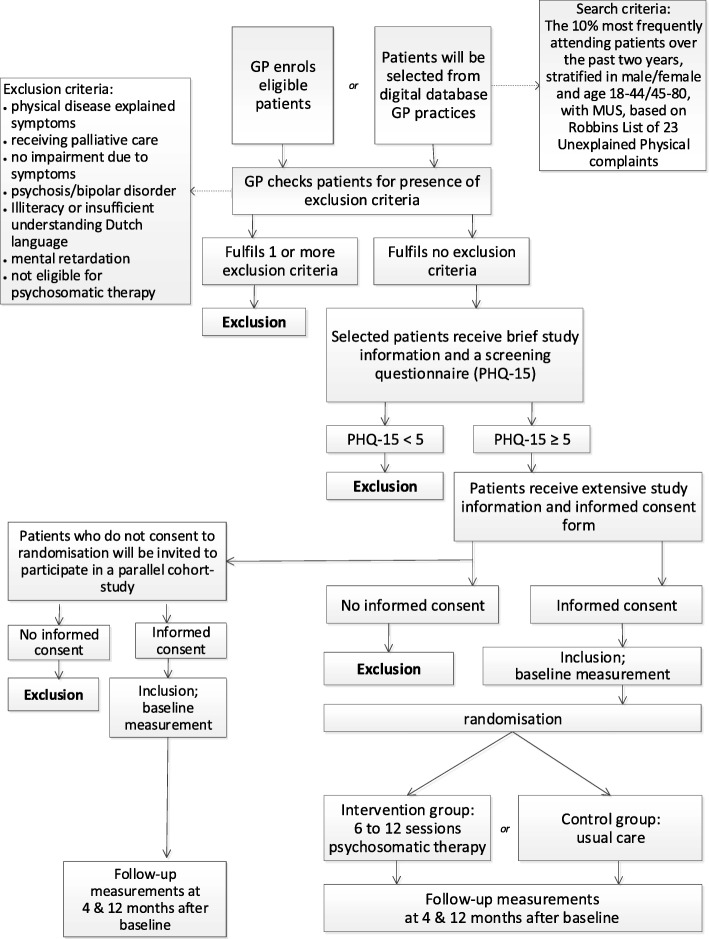
Overview of inclusion procedure. *GP* general practitioner; *PHQ-15* Patient Health Questionnaire

Parallel to the trial, patients who do not consent to randomisation (e.g. due to a strong preference for one of the treatment options) will be asked to complete the same questionnaires, to monitor outcome in this group of patients. The aim of this parallel group is to learn about the differences in patient characteristics at baseline and the course of MUS between the patients in the randomised trial and patients who do not want to participate in the randomised trial in order to better assess the generalisibility of the trial results. In this group of patients no interventions will be carried out on behalf of the investigators, but similar baseline and follow-up measurements will be conducted as in the randomised trial. Figure 2 presents the flow chart of the study with an overview of the inclusion procedure.

Since we consider the psychosomatic therapy to be a complex intervention, we will conduct a process evaluation with quantitative and qualitative methods (mixed-methods) [25]. For the framework of the process evaluation we will use the approach described by Saunders et al., describing five components of process evaluation: fidelity, dose, reach, recruitment and context [26]. The aim of the process evaluation is to explore the mechanism of the intervention, the patients’ experiences with the psychosomatic therapy and understand which patients will benefit most from this therapy.

### Inclusion and exclusion criteria

Patients will be eligible for the study if they are aged 18 years or older, have visited their GP frequently in the last 2 years and have reported one or more ‘Robbins List’ symptoms in the past months [27]. Patients will be excluded from participation in the study if they are older than 80 years, have a Patient Health Questionnaire 15-item somatic symptom scale (PHQ-15) score of < 5 [28, 29], receive palliative care, have a severe psychiatric disorder (i.e. psychosis-related disorders, dementia and bipolar disorder), have a somatic or psychiatric disorder explaining their symptoms, are according to their GP not eligible for psychosomatic therapy or have insufficient understanding of the Dutch language.

Patients can withdraw from the study at any time for any reason without any consequences.

### Treatment arms

#### Psychosomatic therapy

**Fig. 3:**
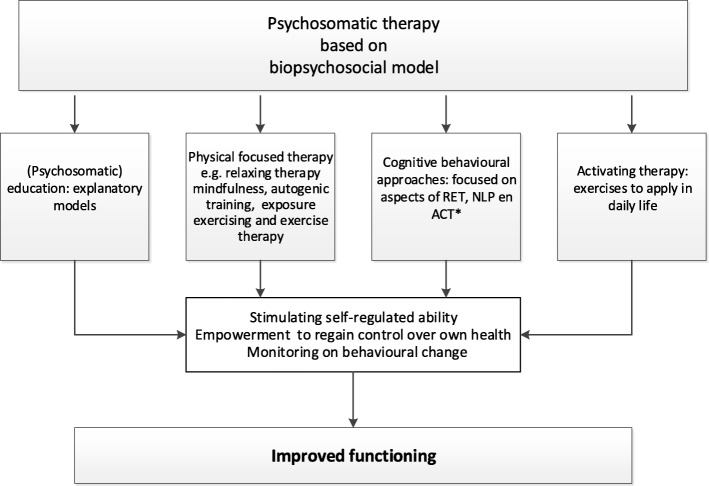
Graphical representation of the assumed working mechanism of psychosomatic therapy. *RET Rational Emotive Therapy*, NLP* Neuro Linguistic Programme*, ACT* Acceptance and Commitment Therapy

Psychosomatic therapy is administered by psychosomatic therapists. These are exercise and physical therapists with a special interest in MUS and registered with the Dutch Association for Exercise Therapists [15] and the Dutch Association for Psychosomatics in Physical Therapy [16], respectively. Psychosomatic therapy has been developed using the well-known concept of the biopsychosocial model in which illness is viewed as a result of interacting mechanisms at the biomedical, interpersonal and environmental levels. It implies that patients’ symptoms, illness beliefs, anxiety, concerns, illness behaviour and social environment are addressed. Psychosomatic therapy is a multi-component, stepped-care and tailored approach and includes the following elements: (1) psycho-education, (2) relaxation therapy and mindfulness, (3) cognitive behavioural approaches and (4) activating therapy. In the psychosomatic therapy sessions the therapist together with the patient explores somatic symptoms and integrates the physical, cognitive, emotional, behavioural and social dimensions of the symptoms presented. During the therapy, underlying beliefs and psychosocial factors which influence the perceived somatic symptoms, are identified in order to give patients (experienced) insight into the interaction of these factors with the somatic symptoms. The aim of the intervention is to improve functioning. The assumed working mechanism of the psychosomatic intervention comprises of three elements, i.e. empowerment of the patients to regain control over their own health, stimulating self-regulated ability and monitoring on behavioural change. Figure 3 presents the graphical representation of these assumed working mechanism of psychosomatic therapy.

For this study a standardised treatment protocol is developed, but the therapists are allowed to change the intensity, frequency and order of the elements in order to deliver personalised care. The psychosomatic therapists will receive an intensive training, an accredited e-learning and two meetings of 4 h each, focussed on MUS, information about the study and the standardised treatment protocol. The patient will not be restricted in seeking other care.

#### Care as usual

Patients in the control group will receive usual care provided by the GP and other health care professionals, with no restrictions. The guideline on the management of MUS of the Dutch College of General Practitioners [30] describes the usual care. It recommends GPs to focus on the exploration of the symptoms, and on related cognitions, emotions and behaviour, and apply psychoeducation, monitoring, and, when necessary, to refer a patient to a physical therapist, a mental health nurse-practitioner or a psychologist.

### Outcome measures

#### Primary outcome

In order to estimate the primary outcome of the treatment we will measure patients’ level of specific functioning and disability measured with the PSFS. The PSFS is a self-reported, thoroughly validated, responsive measurement instrument for up to three individual activities for which patients perceive limitations rated on an 11-point numeric rating scale (NRS) ranging from 0–10 (0 representing ’impossible‘ and 10 ’not a problem at all‘) [24, 31–33]. We will measure mean change from baseline at 4 and 12 months’ follow-ups.

#### Secondary outcomes

Secondary outcomes will be severity of physical and psychosocial symptoms, physical and mental health status, quality of life, health anxiety, illness behaviour and illness beliefs.

Perceived symptom severity is measured on a NRS (range 0–10; 10 represents most severe symptoms) [34].

Patients’ self-rated psychosocial symptoms are measured with the Four Dimensional Symptom Questionnaire (4DSQ). The 4DSQ consists of 50 items with four subscales: distress, depression, anxiety and somatisation. The score for individual items are rated on a five-point Likert scale (no (0), sometimes (1), regularly, frequently or continuously (2)) [35]. Distress and somatisation scores range from 0 to 32 (low: 0–10; moderate: 11–20; high: 21–32), subscale anxiety scores range from 0 to 24 (low: 0–7; moderate: 8–12; high: 13–24) and subscale depression scores range from 0 to 12 (low: 0–2; moderate: 3–5; high: 6–12), higher scores representing worse health.

Physical and mental health status and quality of life are measured with the Short Form Health Survey-36 items (SF-36). SF-36 scores range from 0 to 100, where higher scores correspond to better health [36]. The SF-36 is a widely used, valid and reliable quality of life measure. It consists of 36 items and is categorised into nine subscales: physical functioning, role limitations caused by physical health problems, role limitations caused by emotional problems, social functioning, mental health, energy, pain, general health and health change. These subscales can be summarised into the two summary measures of the SF-36: the mental component summary (MCS) and the physical component summary (PCS) [37].

Health anxiety and illness behaviour are measured with the Illness Attitude Scale (IAS). The IAS, consisting of 29 items, assesses fears, beliefs and attitudes associated with hypochondriasis and abnormal illness behaviour and will be rated on a five-point Likert scale ranging from 0 (never) to 4 (most of the time) [38].

Illness beliefs are measured with the brief Illness Perception Questionnaire (IPQ-B) [39]. A higher score reflects a more threatening view of the illness. The IPQ-B is based on the revised IPQ-R [40] and designed to assess the cognitive and emotional representations of illness. It consists of nine items in total. Six items assess cognitive illness representations: consequences, timeline, personal control, treatment control, identity and comprehension of illness. One item reflects emotional representation, one item assesses a combination of emotional and cognitive representation: concern. The score will be rated on an 11-point scale, ranging from 0 (not at all) to 10 (very much). The last item is an open-question to assess causal representation.

The GP will be asked to report the total number of consultations of the patient in the year after the psychosomatic therapy.

All other medical consumption will be measured with the iMTA Medical Consumption Questionnaire (iMCQ) [41]. This questionnaire measures all relevant health care-related costs like outpatient visits at any medical specialist, hospitalisations and imaging procedures.

Productivity losses due to illness or recovery will be estimated based on patient-reported absences from paid labor measured with the iMTA Productivity Cost Questionnaire (iPCQ) [41, 42]. For all secondary outcome measures we will measure mean change from baseline at 4 and 12 months’ follow-up.

#### Process evaluation

We will conduct a process evaluation with quantitative and qualitative methods (mixed-methods) [25]. The process evaluation will be limited to the patients in the intervention arm and the participated therapists.

##### Quantitative data

Patients of the intervention group will be asked after the last session about their perceived recovery and satisfaction with the psychosomatic therapy. These are measured with the Global Perceived Effect scale (GPE) on a seven-point Likert scale (from completely recovered to worse than ever and from absolutely satisfied to absolutely unsatisfied) [43]. After the first and last sessions of psychosomatic therapy, patients will receive the Session Rating Scale Dutch version (SRS V.3.0). This questionnaire is a four-item visual analogue instrument with four interaction elements. First, a relationship scale rates the session on a continuum from ‘I did not feel heard, understood and respected’ to ‘I felt heard, understood and respected’. Secondly, a goals and topics scale rates the session on a continuum from ‘We did not work or talk about what I wanted to work on or talk about’ to ‘We worked on or talked about what I wanted to work on or talk about.’ The third element requires the participant to rate the session on a continuum from ‘the therapist’s approach is not a good fit for me’ to ‘the therapist’s approach is a good fit for me.’ Finally, and reiterating, the fourth scale looks at how the participant perceives the session in total along the continuum: ‘there was something missing in the session today’ to ‘overall, today’s session was right for me’ [44].

After the first and last sessions of the psychosomatic therapy, the therapeutic alliance will be assessed with the Working Alliance Inventory-Short Form (WAI-SF). The WAI-SF is a shortened version [45] of the Working Alliance Inventory (WAI). It is a widely used 12-item questionnaire that measures the strength of the therapeutic alliance in an ongoing client-therapist interaction. It comprises 12 items that are scored on a five-point Likert scale, ranging from ‘never or rarely’ to ‘very often’ [46].

All participating therapists will be asked to register, per patient, at the end of the treatment: (1) which psychosomatic therapy elements are actually applied; (2) how often these therapy elements are used and (3) the number, timing and start and end date of psychosomatic therapy sessions per patient.

##### Qualitative data

Patients who decline to participate in the study will be asked for their reasons for non-participation. Qualitative data will be collected through interviews by a research team member with 15 to 30 patients, until saturation of the data, in the psychosomatic therapy condition. To examine the patients’ experiences with the psychosomatic therapy, they will be interviewed after the psychosomatic therapy and 1 year later. We will select these patients by applying a purposive sampling strategy taking variation in, e.g. gender, age, region, symptom intensity and symptom interference into account [47]. The first interview will be done face to face within a month after ending the psychosomatic therapy and will focus on the experiences during the psychosomatic therapy and the effects on symptoms. The second face-to-face interview will be conducted a year later and will consist of only two questions: *(1) How are you now?* and *(2) What did you learn from the psychosomatic therapy?* We will encourage the patients to talk freely about their experiences with the psychosomatic therapy and their symptoms. Both interviews will last between 25 and 45 min.

In addition to the interview data, we will use written evaluations of all patients who have attended psychosomatic therapy. After their last session they will be asked to write down what they have learnt.

We will interview all 26 participating therapists after the last session of their first, second or third patient. These interviews examine the therapists’ experiences with the psychosomatic therapy elements and their reasons for choosing specific therapy elements. The questions will focus on (1) which elements are actually applied for which reasons in which patients with MUS according to psychosomatic therapists; (2) what are, according to the psychosomatic therapists, the barriers and facilitators in psychosomatic therapy for patients with MUS. In addition, we will use audio recordings of the sessions to gain understanding in the therapists’ considerations about applying the different elements of the therapy. Each interview will last between 25 and 45 min.

All interviews, both the interviews with the patients as well as the interviews with the therapists, will use a topic list as guidance. The topic list will be developed based on the aim of our study, prior research and patients’ and therapists’ feedback during the CORPUS study. Additionally, to systematically evaluate the experiences with the psychosomatic therapy, we will add topics based on the framework described by Saunders et al. [26]. Based on the topic list, an interview guide with semi-structured, open-ended questions will be formulated and pilot tested in three patients.

All interviews of the participating patients and the therapists will be audio recorded (with consent), transcribed verbatim, anonymised and analysed.

### Participant timeline

An overview of enrolments, assessments and the time of collection within the trial can be found in Fig. 1.

### Sample size and power calculation

The main aim of our study is to establish whether, compared to usual care, psychosomatic therapy improves daily functioning, disability and symptoms, while reducing costs.

Calculations of sample size are based on a minimally relevant difference (mrd) between the two arms of 1 point on the PSFS (range 0–10) [32], with a standard deviation (sd) of 2 points, with an alpha of 0.05 and a power of 0.80. With an estimated dropout rate of 20% after 1 year at least 79 patients per treatment arm need to be included. With this sample size, we can also detect differences in perceived symptom severity (measured on a visual analogue scale (VAS); mrd = 1.3; sd = 2.6) and number of consultations (mrd = 3; sd = 4).

### Recruitment of study participants

The patients will be recruited from general practices participating in the Academic Network of General Practices of the VU Medical Center (ANH-VUmc), the Academic General Practices Network Academic Medical Center (AHN-AMC) and the Radboud University Medical Center Academic General Practices Network (Radboudumc-AHN). If the number of inclusions from these networks of general practices proves to be insufficient we will approach other general practices in the Netherlands (in and around Amsterdam and Nijmegen).

Eligible patients will be identified by a search strategy that selects from the electronic medical records those patients (aged 18 to 80 years) who visited their GP frequently over the past 2 years with MUS, based on a list of 23 unexplained physical complaints composed by Robbins et al. [27]. Patients who visited their GP twice or more in the past months with one or more Robbins List symptoms are eligible. Table 1 presents the Robbins List with 23 physical complaints. The GP will screen the selected list of this frequently attending group patients for potential exclusion criteria and/or other pertinent reasons for not inviting them, such as a terminal disease or insufficient understanding of the Dutch language. In addition to the search strategy, participating GPs will be asked to enrol eligible MUS patients who are not present on the selected list. The identified and screened potential participants not meeting exclusion criteria will be approached by their GP who sends them a package containing a letter with brief information about the study, the PHQ-15, a brief consent form to receive more information about the study and an addressed return envelope. The PHQ-15 is a frequently used and validated questionnaire about physical symptoms [29]. It consists of 15 items, each of which can be scored from 0 (‘not bothered at all’) to 2 (‘bothered a lot’), which results in a total score ranging from 0 to 30. Higher scores indicate higher somatic symptom severity. Scores of 5, 10 and 15 represent cut-off points for low, medium and high somatic symptom severity, respectively [28].

**Table 1:** Symptoms from the Robbins List [27]

1. Back pain	
2. Joint pain	
3. Extremity pain	
4. Headaches	
5. Weakness	
6. Fatigue	
7. Sleep disturbance	
8. Difficulty concentrating	
9. Loss of appetite	
10. Weight change	
11. Restlessness	
12. Thoughts slower	
13. Chest pain	
14. Shortness of breath	
15. Palpitations	
16. Dizziness	
17. Lump in throat	
18. Numbness	
19. Nausea	
20. Loose bowels	
21. Gas or bloating	
22. Constipation	
23. Abdominal pain	

Patients interested in participating in the study, who return the signed brief consent form and the questionnaire, and have a PHQ-15 score of 5 or higher are potentially eligible. They will receive extensive study information, an informed consent form and a return envelope. Patients will be allowed a period of at least 2 weeks to consider their decision on participation in the study. During this period, the researcher will be available to answer any questions that might arise by telephone or e-mail. To participate, the patient will be asked to sign the informed consent form and return it to the researcher. After the signed informed consent form has been received, an e-mail will be sent to the participant with a link to the web-based baseline measurement. In case the patient prefers a paper version of the questionnaires, this will be sent by regular mail.

Patients who do not consent to randomisation (e.g. due to a strong preference for one of the treatment options), will be invited to participate in a parallel cohort study alongside the trial to monitor outcome in this group of patients.

During monthly meetings, the Steering Committee, comprised of two coordinating investigators, the researcher and two methodologists, will monitor the recruitment progress. If the recruitment is suboptimal, the Steering Committee will recommend to approach and add new study sites. The Steering Committee will be responsible for managing all study sites.

According to the local standards of the Medical Ethics Committee of VU University Medical center a data monitoring committee (DMC) is not needed because of the assumed minimal risks of the intervention and short duration of the recruitment and trial. Participating GPs and participating psychosomatic therapists wil remain responsible for the medical situation of their patients. They will report serious adverse events (SAEs) to the coordinating investigators (JCvdW and TCoH). The coordinating investigator (JCvdW) is responsible for reporting SAEs to the accredited METC. SAEs are not expected as the risk of such events as a consequent of participating in this study is extremely low. For above mentioned reasons we also have not planned any interim analyses.

Within the research center, only internal audits will take place. It is not yet known whether our trial will be subject to this.

## Methods: assignment of intervention

### Allocation and blinding

We will use Castor EDC for the online measurements and for randomisation of the participants [48]. Castor EDC uses a variable block randomisation method and randomly assigns participants to one of the two groups, the allocation ratio will be 1:1. The randomisation sequence is masked for study personnel. In order to balance the size of the intervention and usual care groups in each region, randomisation will be stratified according to regions (Amsterdam or Nijmegen). Patients will be included in either the intervention group or the usual care group based on the random allocation. The research assistant will give all patients a unique patient identification number, matching with the corresponding name to inform each participant about the allocation. All patients will be informed about the allocation by the research assistant by regular mail. Patients randomised to the psychosomatic therapy group will also be informed about the psychosomatic therapy by telephone by the research assistant and will receive a referral from their GP to one of the psychosomatic therapists participating in this study. If patients are randomised to the usual care group, they will be offered to receive psychosomatic therapy after completion of the study if still indicated. The research assistant will inform all GPs about the inclusion and allocation of their patients by regular mail or secure e-mail.

Since psychosomatic therapy is a face-to-face intervention, patients and therapists cannot be blinded to allocation. The data analysts will not be blinded to treatment assingments, as this would be unfeasible because they are also involved in data collection.

## Methods: data collection, management and analysis

### Data collection

#### Data collection randomised trial

Data will be collected and stored digitally using Castor EDC [48]. Castor EDC is a Cloud-based Electronic Data Capture platform that enables easily capture of our data. Castor EDC is fully compliant with Good Clinical Practice, maintains stringent data security procedures and provides an audit trail. We will develop a case record form (electronic document designed to record all the information for an individual study subject), build a database, define a procedure for data entry, define and programme validation checks to ensure the consistency of the dataset, and set up the randomisation procedure, web questionnaire and/or logistic module. We will check for data-entry errors and range checks in values weekly to improve data quality during data collection. The participants will receive an e-mail with a link for all baseline and follow-up measurements, and if necessary, a reminder, to complete the measurements online. If participants still fail to respond, an independent research assistant will call them to encourage them to fill out the questionnaires. In case participants prefer a paper version, the measurements will be send by regular mail.

#### Data collection baseline demographic and clinical characteristics

Data will be obtained on patients’ baseline demographic and clinical characteristics by the first questionnaire. The questionnaire consists of questions about age, gender, marital status, socio-economic status (employment and level of education), source of income and working hours. The intensity, duration of the symptoms and interference with daily living, expectations about the prognosis of complaints and about the effect of the treatment will also be assessed in the first questionnaire. The number and severity of the symptoms will be measured with the PHQ-15 [28, 29] (at baseline and at 12 months).

### Data management

Data will be stored on Castor EDC. In case people prefer a paper version of the questionnaires, then the completed paper questionnaires will be stored in a locked closet at the Department of General Practice and Elderly Care Medicine of the VUmc. This data will also be stored on Castor EDC. Participants will be assigned with a code. The code list will be safeguarded by the principal investigator. The principal investigator, researcher, research assistants and coordinating investigator will be able to access the source data. Data will be kept for 15 years.

The audio recordings of all qualitative data will be stored securely in a safety Cloud and only accessible on a password-protected computer. All audio recordings will be destroyed immediately after processing.

### Statistical analysis

The primary data analysis will be performed according to the intention-to-treat principle (ITT) as outlined in the Consolidated Standards of Interventional Trials (CONSORT) Statement [49], i.e. all patients who were enrolled and randomly allocated to either group are included in the analysis and are analysed in the group to which they were randomised. We also will perform a per-protocol analysis. Data of patients in the intervention group who have completed at least four sessions will be used for the per-protocol analysis. Special care will be taken to gather follow-up data on all patients, regardless of whether they comply to the intervention that they are randomised to ,or not.

#### Effect evaluation

##### Effectiveness of the intervention

Differences in the change scores between the intervention group and the control group on the primary and secondary outcomes will be analysed with multilevel (mixed-model) linear regression analyses taking all three measurements into account (baseline, and at 4 and 12 months). We will use a model with a random intercept and all other variables fixed.

A sensitivity analysis will be performed by replacing missing values by multiple imputation. *P* values < 0.05 are considered statistically significant.

Differences in the change scores between the cohort study group and both the intervention group and the control group on the primary, secondary and other study parameters will be analysed with multilevel (mixed-model) linear regression analyses, taking all three measurements into account. We will perform subgroup analyses for severity, duration and number of the symptoms and comorbidity to explore which patients are most likely to benefit from the intervention.

#### Economic evaluation

##### Cost-effectiveness of the intervention

We will conduct an economic evaluation from a societal perspective in accordance with the Dutch guidelines for economic evaluation [50]. Effectiveness is presented in terms of quality-adjusted life years (QALYs). QALYs are a combination of quality of life and survival, where quality of life is expressed as a utility score between 0 (representing death) and 1 (representing perfect health). Utility scores will be calculated through the SF-6D, which is a well-known classification for describing health derived from a selection of SF-36 items [36, 51]. Based on the SF-6D health state, each patient is assigned a utility score at three time points (baseline, 4 months, 12 months), using tariffs from the general population. QALYs will be calculated using the area-under-the-curve method, using the utility scores at baseline, 4 months and 12 months.

Since the economic evaluation will be performed over a time horizon of 1 year, costs and effects will not be discounted.

The cost analysis consists of two main parts. First, at patient level, volumes of care related to the care of patients with MUS will be measured by means of the iMCQ and iPCQ [41, 42]. The second part of the cost analysis consists of determining the cost prices for each volume of consumption. The standard cost prices from the ‘Dutch Guidelines for Cost Analyses’ [52] will be used to determine the cost prices for each volume of consumption. For units of care where no standard prices are available real cost prices will be determined. To obtain total costs, resource use will be multiplied with the cost prices. Productivity losses will be valued by means of the friction cost method [53–55].

Differences in costs and QALYs per patient between the two treatment arms will be calculated, according to the ITT and the per-protocol principle. Patients in the intervention group that have completed at least four sessions will be used for the per-protocol analysis. Where relevant, incremental cost-effectiveness ratios (ICERs) will be calculated to reflect the extra costs per QALY gained.

Uncertainty in the ICER will be presented non-parametrically using bootstrap techniques [56]. The bootstrap replications will be used to estimate 95% confidence intervals around cost and QALY differences. Results will be graphically presented and analysed by means of a cost-effectiveness plane and cost-effectiveness acceptability curves [57]. A secondary analyses will be performed from a health care perspective.

#### Budget impact analysis (BIA)

A budget impact analysis (BIA) will be performed according to the ISPOR Principles of Good Practice for Budget Impact Analysis [58]. Different BIAs will be performed depending on the perceived budget holders; one from a societal perspective and one from the medical perspective. Data from the trial will be used in combination with data that reflect the size of the eligible population, the current mix of treatments and the expected mix after the introduction of psychosomatic therapy, the cost of the treatment mixes, and any changes expected in condition-related costs. Different scenario analyses will be performed in which the input parameters can be varied on plausible ranges of extremes.

#### Process evaluation

##### Analysis of quantitative data

The survey results of participating patients (patients’ perceived recovery and satisfaction (GPE) [43], interaction elements between therapist and patient (SRS-DV) [44] and therapeutic alliance (WAI-SF) [45]) and participating therapists (applied modules, number and period of sessions of psychosomatic therapy) will be presented with descriptive statistics.

##### Analysis of qualitative data

The reasons for patients for not participating in the study will be analysed and coded using MAXQDA 2018 [59] and themes will be identified and described. The process of data collection and analysis will be iterative, the researchers will start with analyse after the first interviews to further explore and validate emerging themes in the next interviews (iterative process). The transcripts will be analysed according to the constant comparison analysis, by two researchers independently, by coding the text using MAXQDA 2018 [59] and themes will be identified and described. Disagreements between the researchers about the themes and codes will be resolved in a consensus meeting. To increase the validity of the transcriptions we will send a summary of the interview to the patients and ask them if they recognise the main themes (member check).

We will report the qualitative research according to the consolidated criteria for reporting qualitative research (COREQ) [60]. We will be making data available to the public on request.

## Discussion

In this randomised controlled trial we will study the effectiveness and cost-effectiveness of psychosomatic therapy for patients frequently attending primary care with MUS. Previous research on the cost-effectiveness of interventions in primary care for patients with MUS was focussed on group-training mindfulness-based cognitive therapy [19, 61], group training with cognitive behavioural techniques [62] or an individual, within the general practice, nurse-led psychological treatment [63]. Our study is the first large-scale pragmatic randomised clinical trial to evaluate the (cost-)effectiveness of an individual, tailored psychosomatic therapy for patients with MUS. Previously, we performed a pilot randomised trial with 36 patients comparing psychosomatic therapy with usual care in order to study feasibility and treatment effects [21]. The pilot study showed that trial retention as well as acceptability of the intervention was good, as 86% of the included patients completed the trial and 81% of the patients were (very) satisfied with the intervention. At 12 months’ follow-up, patients who received psychosomatic therapy showed statistically significant and clinically relevant improvements with regard to perceived symptom severity (adjusted mean difference − 2.0, 95%CI − 3.6 to − 0.3), symptoms of somatisation (adjusted mean difference − 4.4, 95%CI − 7.5 to − 1.4) and symptoms of hyperventilation (adjusted mean difference − 5.7, 95%CI − 10.5 to − 0.8). Almost all outcome measures showed greater improvement in the intervention group than in the usual care group and these were considered clinically relevant [64]. They were all medium to large effects according to Cohen’s statistical guideline with Effect Sizes (ES) [65], calculated with the adjusted differences between the groups, of *d* = 0.79 for perceived symptom severity and *d* = 0.54 and *d* = 0.56 for, respectively, somatisation and health change. Symptoms of hyperventilation had a medium ES of *d* = 0.44. All other outcome parameters did show low ES [21]. However, our pilot, obviously was underpowered so caution is needed in interpreting these effects of treatment.

Although our study is well-considered, the conduction of the study will present potential operational challenges. The first challenge is the inclusion procedure and recruitment of sufficient numbers of patients. To deal with this challenge we will take into account what we have learned in the pilot [21]. Therefore, we will conduct the digital search strategy based on the Robbins List combined with the enrolment by the GP of eligible patients. We will use the PHQ-15 as a screening measurement to objectify the current existence of symptoms of the patients.

The second challenge in this study is the evaluation of the complex intervention and whether the intervention is effective in everyday practice. Therefore, we conduct a process evaluation with quantitative and qualitative methods to understand the whole range of effects and how they possibly vary. In order to establish which components of the intervention will contribute the most to patients’ clinical improvement, we will perform a longitudinal qualitative in-depth interview study alongside the trial. In this part we will evaluate how the intervention works and what are the working elements.

A strength of our study is that the psychosomatic therapy is a well-accessible intervention already delivered in primary care by physical therapists with special interests in MUS. Psychosomatic therapy has an integral approach, focussing on physical, psychological and psychosocial factors; therefore, the intervention is possibly more appropriate for patients who decline CBT. Furthermore, the guideline on the management of MUS of the Dutch College of General Practitioners [30] is very positive about psychosomatic therapy, although the evidence for the effectiveness of this therapy is still lacking. All participating psychosomatic therapists will participate in an intensive training (e-learning and meetings) about the content of the standardised treatment protocol and education in MUS. By this training we will minimise the differences in the psychosomatic therapy delivered by the psychosomatic therapists [66]. In addition, we will conduct a process evaluation through interviews with the participating therapists to explore the possible facilitators and barriers of the psychosomatic therapy from the therapists’ perspective. The findings will improve or refine the intervention. Another strength of our study is the 12-month follow-up measurement as it will result in data being obtained about long-term (cost)-effectiveness of the intervention. The aim of the psychosomatic therapy is to give patients insight into the interaction of their psychosocial factors with their somatic complaints and thus to empower patients to regain control over their own health, focussing on self-management and behaviour changes. Therefore, a 12-month follow-up is appropriate, since the process of behavior change will take at least 6 months [67].

A possible limitation to our study is the exclusion of patients who do not have sufficient understanding of the Dutch language. About 13% of the Dutch population are non-Western immigrants and their offspring. A proportion of them has insufficient command of the Dutch language; therefore, a part of the potential target population for this intervention will probably have to be excluded. Another limitation to our study is that blinding of patients, therapists, GPs and researchers to treatment allocation is not possible, due to the nature of the intervention.

In conclusion, if proven cost-effective, psychosomatic therapy would provide a valuable additional treatment option for adult patients with MUS.

## Trial status

The protocol version number and date: version 10, 8 May 2018. Patient recruitment started in January 2019. The recruitment of participants was ongoing at the time of the submission of this manuscript. Follow-up assessments of patients are expected to be completed in January 2021.

## Supplementary information

**Additional file 1.** Standard Protocol Items: Recommendations for Interventional Trials (SPIRIT) Checklist.

## Data Availability

We will be making data available to the public on request.

## Abbreviations

4DSQ: Four Dimensional Symptom Questionnaire
BIA: Budget impact analysis
CAU: Care as usual
CBT: Cognitive behavioural treatment
FAs: Frequent attenders
GPE: Global Perceived Effect
GPs: General practitioners
IAS: Illness Attitude Scale
ICERs: Incremental cost-effectiveness ratios
iMCQ: iMTA Medical Consumption Questionnaire
iPCQ: iMTA Productivity Cost Questionnaire
IPQ: Illness Perception Questionnaire
MCS: Mental component summary
MUS: Medically unexplained symptoms
NRS: Numeric rating scale
PCS: Physical component summary
PHQ-15: Patient Health Questionnaire 15-item somatic symptom scale
PSFS: Patient-Specific Functional Scale
QALYs: Quality-adjusted life years
SF-36: Short Form Health Survey-36 items
SRS-DV: Session Rating Scale-Dutch version
WAI-SF: Working Alliance Inventory-Short Form
